# The *Salmonella* type III effector SpvC triggers the reverse transmigration of infected cells into the bloodstream

**DOI:** 10.1371/journal.pone.0226126

**Published:** 2019-12-09

**Authors:** Adarsh Gopinath, Taylor A. Allen, Caleb J. Bridgwater, Corey M. Young, Micah J. Worley

**Affiliations:** 1 Department of Biology, University of Louisville, Louisville, Kentucky, United States of America; 2 Department of Microbiology and Immunology, University of Louisville, Louisville, Kentucky, United States of America; Centre National de la Recherche Scientifique, Aix-Marseille Université, FRANCE

## Abstract

*Salmonella* can appear in the bloodstream within CD18 expressing phagocytes following oral ingestion in as little as 15 minutes. Here, we provide evidence that the process underlying this phenomenon is reverse transmigration. Reverse transmigration is a normal host process in which dendritic cells can reenter the bloodstream by traversing endothelium in the basal to apical direction. We have developed an *in vitro* reverse transmigration assay in which dendritic cells are given the opportunity to cross endothelial monolayers in the basal to apical direction grown on membranes with small pores, modeling how such cells can penetrate the bloodstream. We demonstrate that exposing dendritic cells to microbial components negatively regulates reverse transmigration. We propose that microbial components normally cause the host to toggle between positively and negatively regulating reverse transmigration, balancing the need to resolve inflammation with inhibiting the spread of microbes. We show that *Salmonella* in part overcomes this negative regulation of reverse transmigration with the *Salmonella* pathogenicity island-2 encoded type III secretion system, which increases reverse transmigration by over an order of magnitude. The SPI-2 type III secretion system does this in part, but not entirely by injecting the type III effector SpvC into infected cells. We further demonstrate that SpvC greatly promotes early extra-intestinal dissemination in mice. This result combined with the previous observation that the *spv* operon is conserved amongst strains of non-typhoidal *Salmonella* capable of causing bacteremia in humans suggests that this pathway to the bloodstream could be important for understanding human infections.

## Introduction

A key component in the virulence of many successful pathogens is the ability to spread from the initial site of infection to deeper tissue. Following oral ingestion, *Salmonella* can disseminate from the gastro-intestinal (GI) tract to the blood and subsequently internal organs through three independent pathways. In what is believed to be the primary pathway, *Salmonella* as well as numerous other enteropathogens adhere to and invade the M cells of Peyer’s patches and are subsequently internalized by the underlying phagocytes. The infected phagocytes can then migrate to the mesenteric lymph nodes, where they can orchestrate immune responses against the microbes. As *Salmonella*, like many pathogens, can withstand the microbicidal activities of these cells, the bacteria are conventionally thought to passively spread throughout the host after the infected cells drain from the mesenteric lymph nodes through the thoracic duct into the bloodstream. As ingrained as this model is, there is recent evidence, which indicates that the mesenteric lymph nodes act as a firewall, largely containing oral infections, allowing for the generation of a local immune response, while shielding the host from systemic, microbial dissemination[1, 2]. In fact, even though the availability of migratory dendritic cells is the rate-limiting step in mesenteric lymph node colonization, modulation of dendritic cell numbers or migratory properties within the lymphatic system does not affect colonization of the spleen and liver[1]. Moreover, *Salmonella* and *Yersinia* colonize these tissues in mice that completely lack Peyer’s patches with nearly identical kinetics as they do in congenic control mice[2, 3]. Thus, it is curious that passive, ordered dissemination through the lymphatic system to the bloodstream remains the prevailing model to explain the spread of enteropathogens to deeper tissue. In another recently described pathway, *Salmonella* perturbs beta-catenin-dependent signaling in gut endothelial cells, disrupting a gut vascular barrier to gain access to the bloodstream[4].

In an alternative pathway, CD18 expressing phagocytes, presumably dendritic cells, can ferry *Salmonella* directly into the bloodstream, also bypassing the lymphatic system[5]. These cells send processes across the epithelium to engage in intestinal antigen sampling[5]. Normally, after microbe internalization, they presumably mature, become responsive to CCL19 and CCL21 via up-regulation of CCR7 and follow the chemotactic gradients into the lymphatic system. When *Salmonella* enters them however, the infected cells sometimes rapidly penetrate the bloodstream, through an as of yet, largely uncharacterized mechanism. Traversing the blood vascular endothelium in the basal to apical direction is referred to as reverse transmigration. This pathway to the bloodstream is not conventionally thought to enhance microbial virulence[6, 7]. Rather it was proposed that this is a host-controlled process that takes *Salmonella* cells to the spleen, which filters the bloodstream, to engender a systemic immune response against the bacteria to combat subsequent, delayed invasion of deeper tissue through the lymphatic system[6, 7]. We have uncovered evidence however, which suggests that *Salmonella* can actively exploit reverse transmigration to bypass the lymphatic system, expediting its colonization of internal organs[8, 9].

Reverse transmigration is likely relevant to numerous infectious processes including the spread of pathogenic microbes from the GI tract, lung tissue and the oral mucosa to the systemic circulation. Here we report on our studies of how *Salmonella* manipulates reverse transmigration. *Salmonella* infection leads to millions of deaths world-wide every year[10]. *Salmonella enterica* serovar Typhimurium *(S*. Typhimurium) causes gastrointestinal illness in humans, which often is not serious, but can be fatal in infants, the elderly and the immunocompromised. Also, *S*. Typhimurium can sometimes cause bacteremia and even septicemia in otherwise healthy individuals, which is a growing public health threat, especially in immunodeficient people, such as those infected with HIV. *Salmonella* enterica serovar Typhi (*S*. Typhi) on the other hand can cause typhoid fever, a serious systemic illness. *S*. Typhimurium causes a typhoid fever like disease in mice as *S*. Typhi does in humans. In addition to public health concerns, *Salmonella* is also studied because it is a convenient model pathogen.

*Salmonella* harbors two primary pathogenicity islands, termed *Salmonella* pathogenicity island one (SPI-1) and *Salmonella* pathogenicity island two (SPI-2). SPI-1 is used to invade host cells and invoke an inflammatory response and can kill host cells[11–14]. SPI-2 on the other hand promotes intracellular growth[15–17] and we have previously demonstrated that it is required for the rapid appearance of infected cells in the bloodstream following oral inoculation of mice[8, 9]. We showed that one allele of the SPI-2 associated type III effector SrfH/SseI accelerates the appearance of *Salmonella*-infected cells in the bloodstream potentially through an interaction with the host protein TRIP6[9].

Some *Salmonella* serovars carry plasmids, which share a highly conserved locus called the *spv (Salmonella* plasmid virulence) operon[18]. It has been suggested that *spv* genes are important for human pathogenesis as *spv*-carrying strains dominate among clinical isolates from patients with non-typhoidal bacteremia[19, 20]. SpvC is a phosphothreonine lyase that dephosphorylates Erk1/2, p38 and JNK[21, 22]. An *spvC* mutant is not defective in replication within macrophages but is attenuated in mice[21].

Here, we describe the development of an *in vitro* reverse transmigration assay to model the rapid colonization of the bloodstream by *Salmonella*-infected cells following oral ingestion. We demonstrate that microbial components down regulate reverse transmigration and that *Salmonella* overcomes this by secreting SpvC and at least one additional unknown SPI-2 associated type III effector into infected dendritic cells that stimulate reverse transmigration. These results suggest that the reverse transmigration pathway to the bloodstream could be an important component of *Salmonella* pathogenesis.

## Results

### Development of an *in vitro* reverse transmigration assay

**Fig 1:**
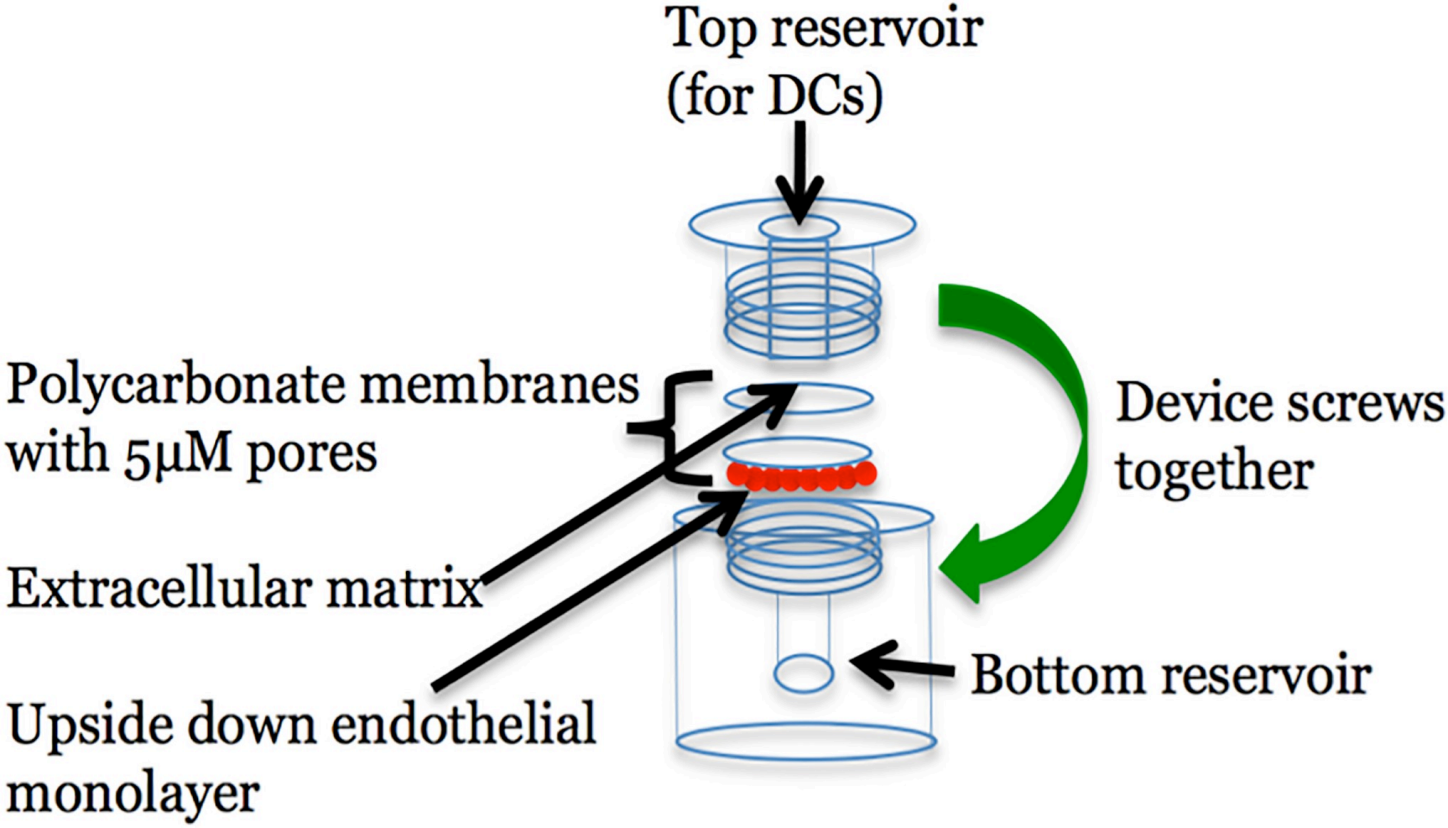
An *in vitro* reverse transmigration assay. In our *in vitro* reverse transmigration assay, two PVP-free polycarbonate membranes with 5μm pores are sandwiched in between the upper and lower compartments of a blind well. Media is placed in the bottom compartment and then the bottom membrane is placed over the liquid which contains an upside-down confluent monolayer of endothelial cells. The upper membrane is then placed on top of the bottom membrane right side up. It is coated with extracellular matrix. The device is then screwed together and dendritic cells are added to the upper compartment. The devices are incubated at 37°C for one hour. Then the liquid is carefully aspirated from the top compartment, the device disassembled and the membranes discarded. The media from the bottom compartment is withdrawn and reverse transmigration measured by either counting uninfected dendritic cells with a hemocytometer or lysing infected dendritic cells and recovering CFU on agar plates.

**Fig 2:**
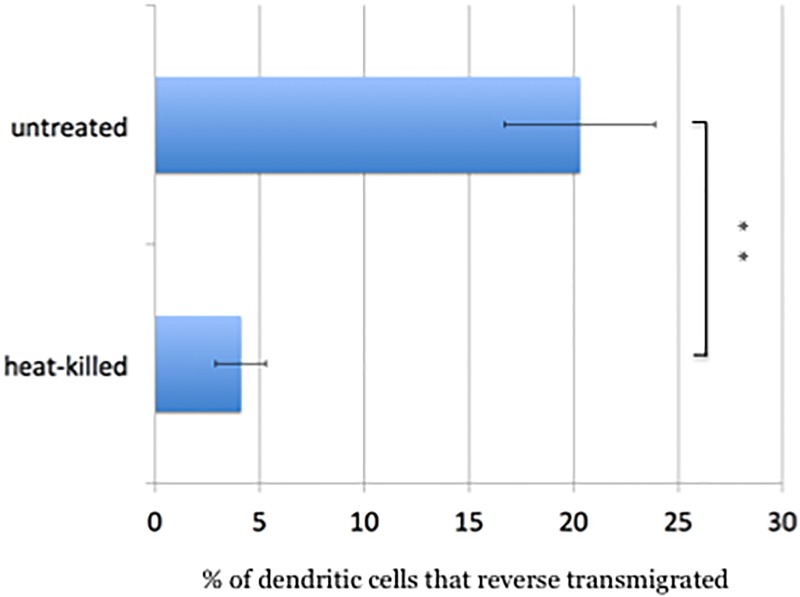
Bacterial components inhibit reverse transmigration. Dendritic cells were treated with either LB or LB containing heat-killed *S*. Typhimurium and the reverse transmigration assay performed. Heat-killed bacteria decreased reverse transmigration five-fold. This experiment was performed in triplicate on three different occasions. Error bars represent the standard deviation. ** p-value <0.01.

We used blind wells to establish an *in vitro* reverse transmigration assay adapted from Bianchi *et*. *al*.[23] that models how dendritic cells can reenter the bloodstream in the basal to apical direction (Fig 1). Blind wells consist of two compartments that you can sandwich membranes in between. In our assay, C166 murine endothelial-like cells were grown into monolayers on PVP-free polycarbonate membranes with 5μm pores. The formation of confluent monolayers was confirmed with diff-kwik staining. One membrane was placed upside-down over the top of the bottom compartment of the blind well, which was filled with media. Another membrane with a monolayer of cells was stripped, revealing a natural extracellular matrix and was placed right side up on top of the first membrane. The device was then screwed together. Murine bone marrow-derived cells were differentiated into dendritic cells with GMCSF, IL-4 and TGF-ß. The cytokine treatment routinely produced a heterogenous population of cells of which 30%-40% were CD11c^+^, presumably a mixture of macrophages and dendritic cells. Dendritic cells are the only cell type capable of reverse transmigration[24]. After seven days of cytokine treatment, we added the cells to the top compartment and after one hour of incubation, the device was disassembled and the liquid withdrawn from the bottom compartment and dendritic cells were counted with a hemocytometer. 100,000 cells were added to the top compartment and in one hour, about 20% had reverse transmigrated through the endothelial monolayer (Fig 2).

### The presence of bacteria deters reverse transmigration

The rapid appearance of *Salmonella* in the bloodstream following oral ingestion was initially proposed to be passive on the part of the bacteria[7]. However, it seems as though the host would have a vested interest in denying bacteria access to the bloodstream and deeper tissue. Accordingly, we tested whether heat-killed *Salmonella* might deter the reverse transmigration of dendritic cells. We incubated cells with or without heat killed *Salmonella* for thirty minutes and then applied them to the endothelial monolayers in the blind wells. After one hour of incubation, we disassembled the blind wells and enumerated the number of dendritic cells that migrated through the endothelial monolayer into the bottom compartment with a hemocytometer. Treating the dendritic cells with dead *Salmonella* inhibited reverse transmigration five-fold (Fig 2).

### SPI-2 stimulates reverse transmigration

We next tested whether or not live *Salmonella* could up-regulate reverse transmigration. In these experiments we infected dendritic cells with either wild type bacteria, a SPI-2 structural mutant that could not secrete any SPI-2 effectors, or one with a transposon disruption in *srfH*. We deleted *sipB* from all three strains as it was previously reported to kill dendritic cells[14]. In agreement with this report, in initial experiments using a wild type background, we observed that *Salmonella* killed some of the dendritic cells (unpublished observation). We chose to perform the experiments with strains lacking *sipB* simply to reduce the number of dendritic cells required for our assays. In subsequent mouse experiments, we did not find it necessary to use the *sipB* mutant background. The inclusion of gentamicin in our *in vitro* assay selectively killed the extracellular bacteria. We infected dendritic cells separately with the three strains and incubated them for six hours prior to performing the reverse transmigration component of the assay. While *in vivo*, SPI-2 associated genes can be expressed in as little as 15 minutes post-infection prior to penetrating the intestine, *in vitro*, in cell culture models of infection, it takes four hours for them to be induced and expression peaks at six hours post-infection[25]. Unlike the experiments with uninfected dendritic cells, we could not count the number of dendritic cells that reverse transmigrated with a hemocytometer because the majority of the cells were not infected and the uninfected ones would also reverse transmigrate and dilute the phenotypes. Accordingly, in order to specifically look at infected dendritic cells, we lysed the dendritic cells with dilute detergent and recovered CFU on agar plates. We tried to detect reverse transmigrating cells infected with strains of bacteria expressing the green fluorescent protein but found it was not feasible to scale up to the point where we had enough reverse transmigrating cells to enter the linear range of a flow cytometer. CFU is actually more informative anyway since it readily distinguishes between live and dead bacteria. Prior to adding the infected dendritic cells to the blind wells, we lysed a small aliquot and plated for CFU to determine the input. After the reverse transmigration assay, we lysed the host cells present in the bottom compartment and similarly recovered CFU on agar plates. In control experiments we did survival assays on the three strains within dendritic cells for the entire length of the assay (including the one hour they were in the blind wells) and observed no differences (S1 Fig), indicating that differences in CFU recovered with the three strains was not due to differences in persistence.

**Fig 3:**
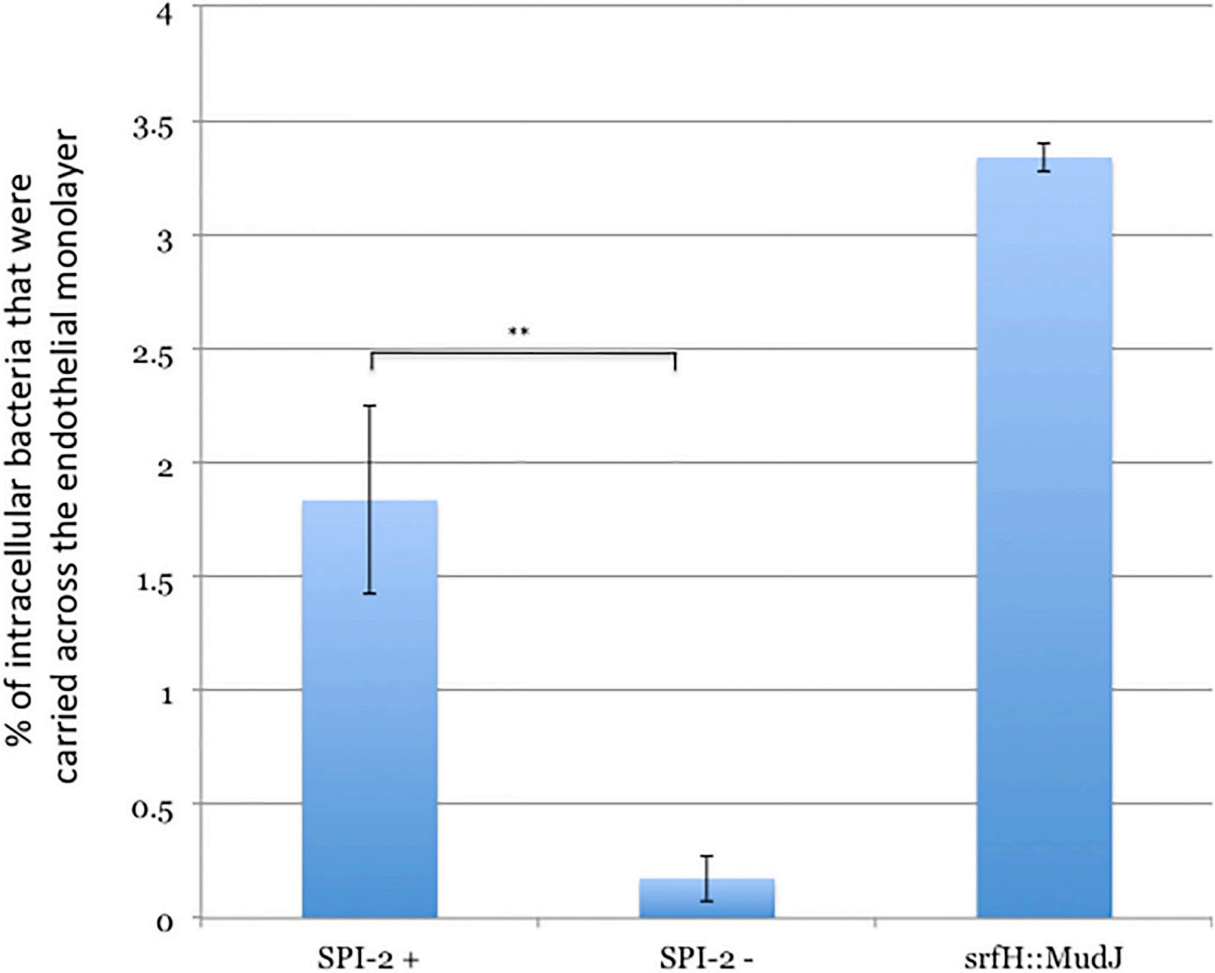
SPI-2 stimulates reverse transmigration. Dendritic cells were infected with either SPI-2 + or SPI-2 –bacteria or a *srfH* mutant. Surprisingly, the *srfH* mutant had no defect in triggering reverse transmigration and in fact was a little more efficient at it than wild type but the difference was not statistically significant. The strain defective in all SPI-2 secretion on the other hand triggered reverse transmigration over an order of magnitude less efficiently than wild type. This experiment was performed in quadruplicate on two independent occasions. Error bars depict the standard deviation. ** p-value <0.01.

We were unable to detect a defect in our reverse transmigration assay for the *srfH* mutant, which we previously reported has about a five-fold defect in penetrating the bloodstream of mice following oral infection[8] (Fig 3). In fact, the *srfH* mutant was more efficient than wild type at triggering reverse transmigration, although the difference was not statistically significant. We observed a very large 10.5-fold defect however in our reverse transmigration assay for the mutant that could not secrete any SPI-2 effectors (Fig 3). This result indicated that *Salmonella* actively manipulates reverse transmigration by secreting at least one type III effector into infected dendritic cells that stimulates the process. We cannot rigorously exclude the possibility that the monolayers lost confluence during the course of the assay as there is no way to get an electrode into the bottom compartment of an assembled blind well. However, this possibility seems very unlikely because if the dendritic cells were going through holes in the monolayer, you would expect them to go through at roughly the same rate, regardless of what strain of bacteria they were infected with. Regardless, we excluded the possibility that the dendritic cells were getting though holes in the monolayer with FITC-labeled dextran beads. In S2 Fig, we demonstrate that greater than 99.9% of the beads are excluded from the bottom compartment of a blind well by the endothelial monolayers. Even though we killed the extracellular bacteria with gentamicin prior to adding the infected dendritic cells to the blind wells, as an additional control we incubated eight blind wells with 10,000 non-invasive *Escherichia coli* cells and observed that the endothelial monolayers excluded greater than 99.9% of the bacteria from the bottom compartment. Cumulatively, it seems safe to conclude that the vast majority of the bacteria that we recovered from the bottom compartment of the blind wells in our *in vitro* reverse transmigration assays were carried there by reverse transmigrating dendritic cells. Even if a minority of the dendritic cells got through holes in the monolayer this would only cause us to underestimate the magnitude of our phenotypes and would not actually alter our conclusions since again, presumably dendritic cells would get through holes in the monolayer at the same rate regardless of what strain of bacteria they were infected with.

### SpvC stimulates reverse transmigration *in vitro*

**Fig 4:**
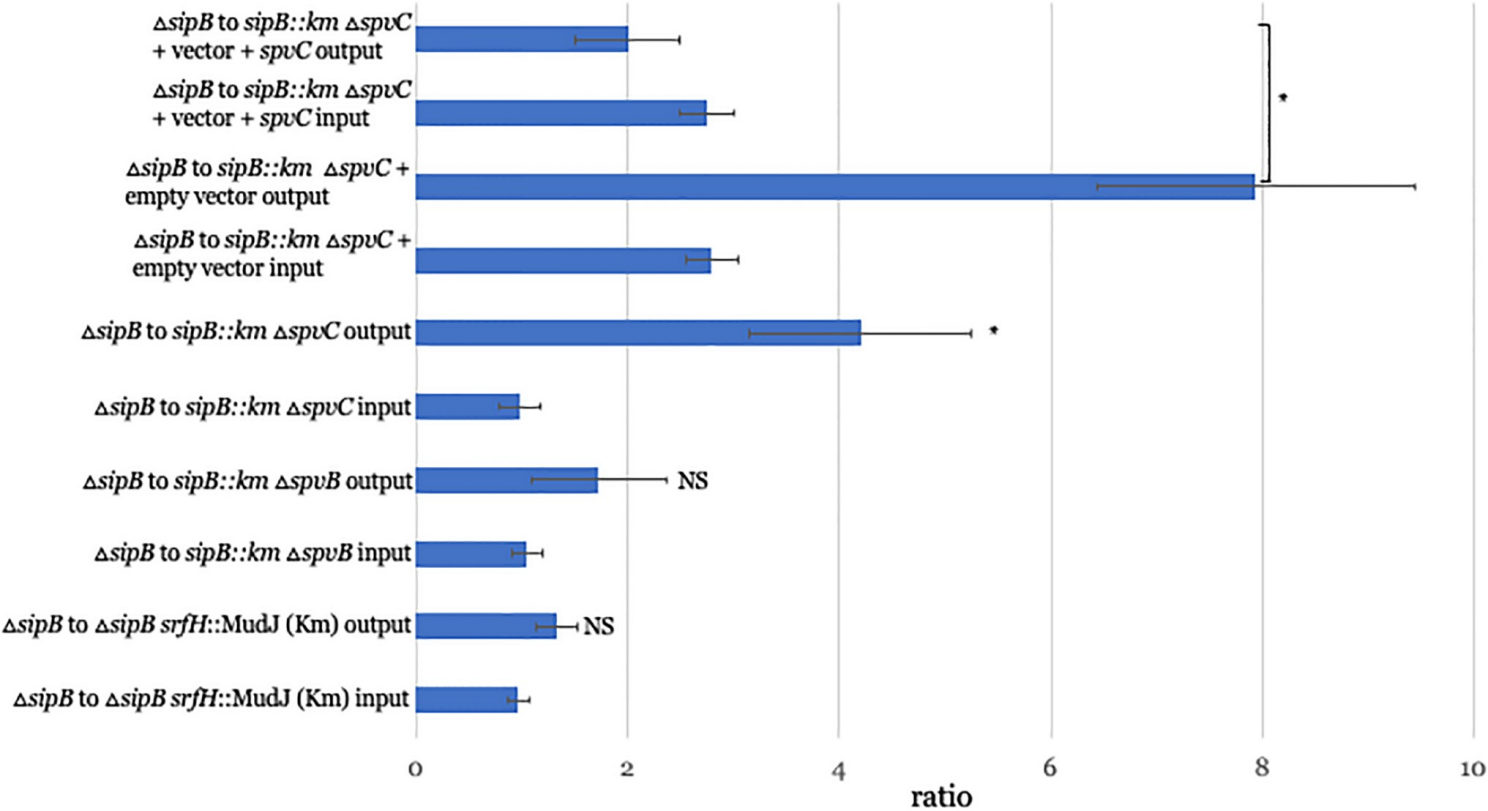
*spvC* triggers reverse transmigration in a competition assay. Dendritic cells were separately infected with wild type bacteria or a kanamycin-resistant mutant. After six hours, the two populations of infected cells were combined and a small aliquot lysed and CFU recovered on LB versus LB-kanamycin plates to determine the input ratio, which was always close to 1:1. The remainder of the mixture was added to the top compartment of a blind well and incubated. After one hour, the blind well was disassembled and the infected cells in the bottom compartment lysed and CFU recovered again on LB versus LB-kanamycin plates. In this competition assay, *srfH* and *spvB* had a negligible effect. Δ*spvC* however displayed a 4.2-fold defect. Five replicates of each competition assay were performed on four independent occasions. Error bars depict the standard error of the mean. *p-value <0.05.

In order to detect a potentially subtle effect of SrfH on reverse transmigration, we developed a competition assay that took advantage of the fact that the *srfH* mutant as well as the other ones we tested are resistant to the antibiotic kanamycin. In these experiments, we infected cells separately with either wild type *Salmonella* or a mutant, killed the extracellular bacteria with gentamicin and combined them and plated an aliquot onto Lura-Bertani (LB) agar and onto LB agar supplemented with kanamycin to determine the input ratio. We then performed the reverse transmigration assay with the remainder of the mixture and plated the output onto LB agar and LB agar augmented with kanamycin. Surprisingly, even in this assay, we could detect no effect of SrfH on reverse transmigration (Fig 4).

We next turned our attention to the *spv* operon, which was reported to be conserved amongst strains of non-typhoidal *Salmonella* that cause bloodstream infections of humans[20]. We first tested an in-frame deletion of *spvB* as SpvB was reported to depolymerize actin[26–28] and thus could potentially play a role in facilitating reverse transmigration. Surprisingly, as with SrfH, SpvB had a negligible effect on reverse transmigration.

We proceeded to test an in-frame deletion of *spvC*, as SpvC was reported to deactivate signal transduction pathways whose activation might discourage reverse transmigration. An *spvC* in-frame deletion mutant displayed a 4.2-fold defect versus wild type in a reverse transmigration competition assay (Fig 4). We were able to complement the phenotype with ectopic expression of SpvC in the mutant background.

### SpvC promotes early extra-intestinal dissemination

**Fig 5:**
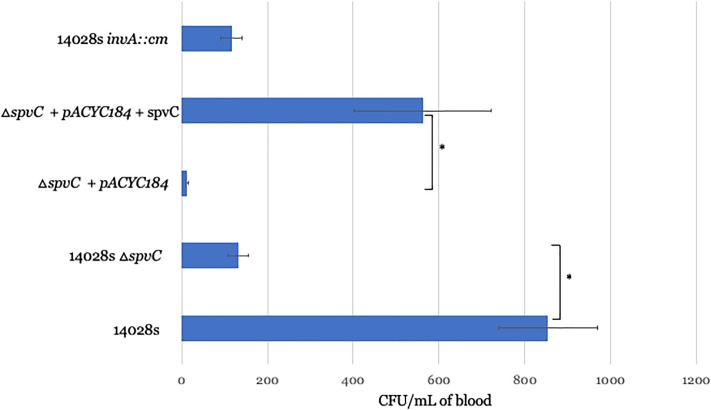
*spvC* triggers reverse transmigration in mice. Groups of 5–8 mice were orally inoculated by gavage with the indicated strains and peripheral blood recovered by heart puncture 30 minutes later. Δ*spvC* displayed a 6.5-fold defect versus wild type *S*. Typhimurium. The phenotype complemented with ectopic SpvC expression from a plasmid. In contrast, to the *in vitro* assay, *in vivo*, SPI-1 appears to enhance reverse transmigration, presumably by facilitating the invasion of the dendritic cells associated with the GI epithelium. These infections were performed on at least three independent occasions. Error bars depict the standard error of the mean. *p-value <0.05.

We orally infected mice with either wild type *Salmonella* or a derivative that differed only in containing an in-frame deletion of *spvC* and thirty minutes later withdrew peripheral blood. We and others have previously shown that all blood-borne *Salmonella* at this time point are within CD18-expressing phagocytes. In fact, no *Salmonella* can be recovered from the bloodstream of CD18 deficient mice at 30 minutes post-infection[7, 8]. CD18 is one of the host molecules required for reverse transmigration[24]. We lysed the host cells with detergent and recovered *Salmonella* on XLD-agar plates. Consistent with the *in vitro* experiments, we observed a 6.5-fold defect for an *spvC* deletion in early travel from the GI tract to the bloodstream (Fig 5). The phenotype complemented with plasmid-borne expression of SpvC in the mutant background (Fig 5). We also tested a SPI-1 mutant to explore how *Salmonella* overcomes SPI-1 mediated killing of dendritic cells. Surprisingly, impairing SPI-1 significantly reduced reverse transmigration suggesting that it functions differently *in vivo* than it does *in vitro*. Under our *in vitro* conditions SPI-1 seemed predisposed towards killing the dendritic cells and impairing reverse transmigration. *In vivo*, however, SPI-1 appears to enhance reverse transmigration, presumably by facilitating the invasion of the dendritic cells associated with the GI epithelium (Fig 5).

## Discussion

This study identifies reverse transmigration as the likely process responsible for the rapid appearance of *Salmonella*-infected cells in the bloodstream of mice following oral infection that we previously observed[8, 9]. Our results indicate that dendritic cells down regulate reverse transmigration in the presence of bacteria and that *Salmonella* in part overcomes this by secreting at least two SPI-2 effectors into infected cells that encourage reverse transmigration. Enhancing our understanding of reverse transmigration is medically important for a variety of reasons. This process likely plays a role in pathologic conditions including the invasion of the bloodstream by cancerous cells and the resolution of excessive inflammation in addition to the inadvertent dissemination of intracellular pathogens from infected tissue into the bloodstream. This inadvertent dissemination may not only be from the GI tract to the blood, but may also play a role in numerous infectious processes including the spread of pathogens from lung tissue and the oral mucosa to the systemic circulation[29, 30]. The potential role of reverse transmigration in cancer and infectious diseases raises the possibility of designing drugs that inhibit the process. The corollary is that drugs, which augment the process, might be useful in cases of chronic inflammation such as autoimmune or graft versus host disease. Remarkably, for all of the implications for human pathological processes, reverse transmigration is very poorly understood.

It is interesting to consider why *S*. Typhimurium seeds internal organs quickly. The speed with which the bacteria penetrate the liver and gall bladder of its animal reservoir may be a component of its virulence. These are privileged sites of infection that are rich in nutrients and generally free of endogenous flora and thus can support the extensive replication of *Salmonella*, before the bacteria return to the GI tract through the lymphatics (connected to the liver) or the bile duct (connected to the gall bladder) for extra-host dissemination. The gall bladder is an extremely beneficial niche for *Salmonella* as 2–6% of the time, the bacteria become asymptomatic here and can potentially be shed intermittently for the lifetime of the host[31–34]. As *Salmonella* infections are often, however, ultimately resolved by an adaptive immune response[32], the bacteria may be under time pressure to reach these organs as quickly as possible, and in essence, be in a race with the immune system. As penetrating the bloodstream through the lymphatic system can take days[35, 36], manipulating reverse transmigration may be a clever strategy through which *Salmonella* bypasses mesenteric lymph node confinement, accelerating its intra-host dissemination. This may increase the transmission rate by providing the bacteria with additional time to grow in their preferred sites of replication and also with more opportunities to establish a chronic, asymptomatic infection. *spvC* is found in non-typhoidal strains of *Salmonella* and is likely a virulence factor in its animal reservoir where it does cause systemic disease. The fact that it can also rapidly colonize the bloodstream of humans where it does not typically cause systemic disease may be unintentional on the part of the bacteria.

The pathway is inefficient at 30 minutes post-infection anyway with only about 1 in a million inoculated bacteria being translocated from the GI tract to the bloodstream in this time-frame. Reverse transmigration could still play an important role in *Salmonella* pathogenesis however as the bacteria presumably disseminate to the bloodstream from the GI tract through this pathway throughout the course of infection. Also, very few *S*. typhi founder cells are needed to seed the spleen and liver to cause problems as they can grow extensively there and any non-typhoidal *Salmonella* organisms in the bloodstream can potentially cause health problems. The fact that the *spv* operon is conserved among non-typhoidal *Salmonella* strains that cause bacteremia in humans further suggests that this pathway to the bloodstream could be important for understanding human infections.

It is interesting to consider potential molecular mechanisms underlying the ability of SpvC to promote reverse transmigration. SpvC deactivates, Erk1/2, p38 and JNK[21, 22]. One possible mechanism among many is that targeting the JNK pathway abrogates endothelin signaling. Endothelin is a ligand produced by vascular endothelial cells that upon binding its G protein-coupled receptor transduces a signal through the JNK pathway that discourages migration[37–39]. Endothelial cells likely secrete endothelin to discourage metastasis and perhaps also to regulate reverse transmigration.

It was surprising that we did not observe a defect in reverse transmigration for dendritic cells infected with a *srfH* mutant, as we have shown in the past that *srfH* is involved in the early penetration of the bloodstream by *Salmonella*-infected cells following oral inoculation of mice[8, 9]. It is possible that SrfH is involved in a step prior to reverse transmigration. Perhaps SrfH is involved in disassociating the dendritic cells from the GI epithelium before the cells become available for reverse transmigration. It is also of course possible that our model does not capture everything that occurs during reverse transmigration *in vivo*.

Our results demonstrate that dendritic cells down regulate reverse transmigration in the presence of microbial components and that *Salmonella* in part overcomes this inhibition by secreting at least two SPI-2 effectors into them that stimulates the process by over an order of magnitude. This work provides some suggestive evidence that the CD18 expressing phagocyte pathway to the bloodstream involves reverse transmigration and that this may be an important component of the extraintestinal dissemination of *Salmonella*. The model described here may be useful in studying the dissemination of other pathogens and could also be used for studying metastasis and excessive inflammatory disorders.

## Materials and methods

### Ethics statement

Animals were housed, cared for, and used strictly in accordance with the USDA regulations and the NIH guide for the care and use of laboratory animals (NIH publication no. 85–23, 1985). The University of Louisville is fully accredited by the American Association for the Accreditation of Laboratory Animal Care. A full-time, specialty-trained veterinarian directs the program of animal care. The protocol was approved by the University of Louisville Institutional Animal Care and Use Committee (protocol # 12–090). All reasonable efforts were made to alleviate discomfort.

### Mice, cell culture and bacterial strains

Six to eight-week-old female C57BL/6J mice were obtained from Jackson labs. (Bar Harbor, ME). Bone marrow was harvested and monocytes cryopreserved as previously described[40].

For extra-intestinal dissemination assays, groups of 5–8, 4–6 week-old female C57BL/6J mice were orally infected by gavage with 1 X 10^9^ bacterial cells suspended in 100μl of phosphate buffered saline (PBS). Food was withdrawn 12 hours prior to infection. Thirty minutes following infection, mice were euthanized with CO_2_ and blood recovered by heart puncture with a 25G needle attached to a 1mL syringe. Blood was collected in microtubes on ice containing 50 units of heparin sodium salt (Sigma) in 100μl of water to prevent coagulation. Triton X-100 was added to a final concentration of 1% to lyse host cells and the tubes incubated at 4°C with end over end rotation on a rotisserie for ten minutes. CFU were then recovered on xylose lysine deoxycholate agar plates, which are selective for *Salmonella*.

C166 murine endothelial-like cells were cultured in DMEM (VWR) supplemented with 10% FBS (Sigma) and sodium pyruvate (Life technologies) and passaged 1:10 every 4–5 days. Monocytes were cultured at a concentration of 1 X 10^6^ cells/mL in RPMI supplemented with 10% FBS and sodium pyruvate. They were differentiated into dendritic cells by culturing them in the presence of GM-CSF (Life technologies) (20ng/mL) and IL-4 (Life technologies) (20ng/mL) for three days. The media also included 55μM ß-mercapoethanol (Life technologies). On the third day, an equal volume of media supplemented with fresh GMCSF (40ng/mL), IL-4 (40ng/mL) and 110μM ß-mercapoethanol was added. On the sixth day, TGF-ß1 (R&D systems) was added to a final concentration of 10ng/mL to induce expression of CD16, which is associated with an enhanced ability to reverse transmigrate[41]. Assays were performed 24 hours later.

*S*. Typhimurium 14028s with an in-frame deletion of *sipB* described in Kidwai et. al.[42] was the parent strain for testing the effects of SPI-2 and *srfH*. We separately transduced with P22 HT int a *srfH*::MudJ allele[43] into this background and an *ssaK*::*km* allele[44] with established techniques[45]. *ssaK* is part of an operon of structural genes that compose the type III secretion system. This mutant cannot secrete any effectors associated with SPI-2[44]. Into previously described strains that separately contained in frame deletions of *spvB* and *spvC*[42], we transduced a previously described *sipB*::Km allele[42]. *spvC* was PCR amplified from the virulence plasmid of *S*. Typhimurium 14028s and cloned into the EcoRV and SalI sites of pACYC184, under the control of the constitutive tet promoter. The construct was sequence verified.

### Growing endothelial cells on membranes

PVP-free polycarbonate membranes with 5μm pores were sterilized by autoclaving and individual membranes were submerged in 1mL of PBS (Life technologies) supplemented with 8μg of fibronectin from bovine plasma (Sigma) in 24 well plates. The membranes were coated with fibronectin overnight at 4°C. The following day, the fibronectin solution was aspirated from the wells and 5 X 10^5^ endothelial cells were added to each well. The endothelial cells were given 5 days to form confluent monolayers. Twenty-four hours before assays began, endothelial cell media was replaced with fresh media supplemented with TNFα (Life technologies) (20ng/mL). The formation of confluent monolayers under these conditions was confirmed with diff-kwik staining.

### Infections and individual reverse transmigration assays

The three bacterial strains were grown overnight at 37°C in LB. Approximately 1 X 10^6^ dendritic cells were infected at an MOI of 25 in quadruplicate with the three bacterial strains. The bacteria were given one hour to invade. Under the conditions used, 1–2% of the bacteria were internalized. Next, gentamicin (Life technologies) was added to a final concentration of 100μg/mL to selectively kill the extracellular bacteria and the cells were incubated for one hour at 37°C. After the one-hour incubation the mixture was dialyzed against PBS with Slidalyzer mini dialysis devices with a 2kDa molecular weight cutoff (Thermo Fischer). Following dialysis, the mixture was incubated in media supplemented with 10μg/mL gentamicin for five hours at 37°C. After five hours, the mixture was again dialyzed against PBS and a small aliquot lysed with 1% triton X-100 for ten minutes and CFU recovered on agar plates to determine the input.

Two-hundred microliters of media containing gentamicin was added to the bottom compartment of the blind wells. Then, a membrane coated with a confluent monolayer of endothelial cells was placed upside down in the device. A second membrane was then stripped by dipping it into a solution of PBS containing 0.5% triton X-100 and 20mM ammonium hydroxide (Sigma) for 30 seconds and then rinsed in DMEM, revealing a natural extracellular matrix[23]. This membrane was placed right side up on top of the first membrane. The device was then screwed together and the infected dendritic cells added to the top compartment. The blind wells were incubated at 37°C for one hour. Then, the liquid in the upper compartment was aspirated, the devices were disassembled, the membranes discarded and the media in the bottom compartment withdrawn and dialyzed against PBS as described for the input. The dendritic cells were then lysed in 1% triton X-100 and CFU recovered on agar plates.

### Reverse transmigration assay with heat killed bacteria

In this experiment, an equal volume of either LB or a saturated LB overnight culture of *S*. Typhimurium was diluted 100-fold in cell culture media and heated to 95°C for 15 minutes. 1 X 10^5^ dendritic cells were then resuspended in either the LB or the heat-killed *Salmonella* and incubated at 37°C for 30 minutes. The dendritic cells were then counted on a hemocytometer to determine the input and applied to the blind wells. They were processed as described above except instead of recovering CFU, the number of dendritic cells that traversed the endothelial monolayer was determined by concentrating the media in the bottom compartment and counting the cells with a hemocytometer.

### Competition assays

1 million dendritic cells were infected with either wild type bacteria or a *srfH*, *spvB*, or *spvC* mutant. The infections were processed as described for the individual assays. After the five-hour incubation, the wild type bacteria and one of the mutants were combined and a small aliquot lysed and CFU recovered on LB-agar plates or LB-agar plates supplemented with 60μg/mL kanamycin to determine the input ratio. The reverse transmigration assay was then performed with the remainder of the mixture as described for the individual assays except after lysis of the cells in the bottom compartment, CFU were recovered on LB-agar plates or LB-agar plates supplemented with kanamycin. The number of ÇFU present on kanamycin plates was subtracted from the number present on LB plates to determine how many wild type bacteria were present. The CFU counts of the kanamycin plates revealed how many mutant bacteria were present.

### Survival assay

In this experiment, 1.7 X 10^5^ dendritic cells were infected at an MOI of 25 with the three bacterial strains and a gentamicin protection assay performed. The number of intracellular CFU at seven hours post-infection was determined by dialyzing the gentamicin and lysing the dendritic cells with triton X-100 and recovering bacteria on agar plates.

## Supporting information

S1 Fig The three strains survive similarly within dendritic cells over the course of the assay. Dendritic cells were infected separately with the three strains and a gentamicin protection assay performed. This assay was performed in triplicate on two independent occasions. There was no significant difference in the number of bacteria present with the different strains at seven hours post-infection.
(TIFF)

S2 Fig On three occasions, blind wells with endothelial monolayers were incubated for one hour with 250μg/mL of FITC-labeled dextran beads (MW 70,000) and the fluorescence of the bottom compartment determined. The monolayers excluded greater than 99.9% of the beads.
(TIFF)
