# Supplementary figures and images for: The Salmonella type III effector SpvC triggers the reverse transmigration of infected cells into the bloodstream
Source: PLoS One. 2019 Dec 9;14(12):e0226126. doi: 10.1371/journal.pone.0226126 (PMC6901223; doi:10.1371/journal.pone.0226126)

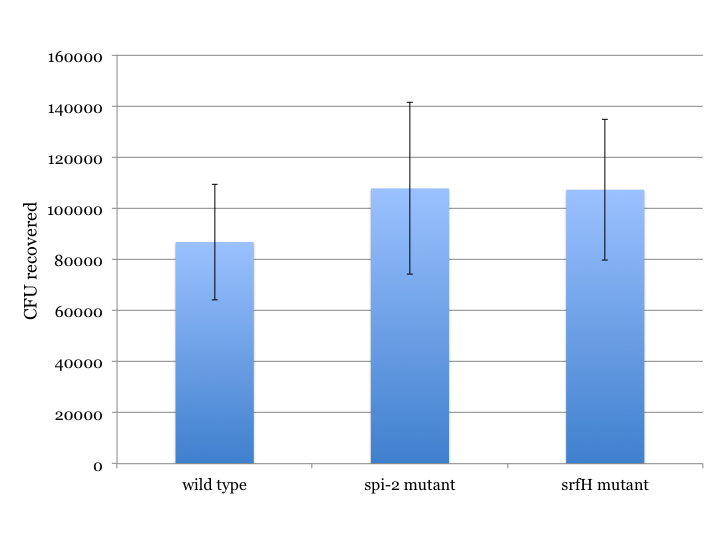

Supplement: S1 Fig — Dendritic cells were infected separately with the three strains and a gentamicin protection assay performed. This assay was performed in triplicate on two independent occasions. There was no significant difference in the number of bacteria present with the different strains at seven hours post-infection. (TIFF) [file pone.0226126.s001.tiff]

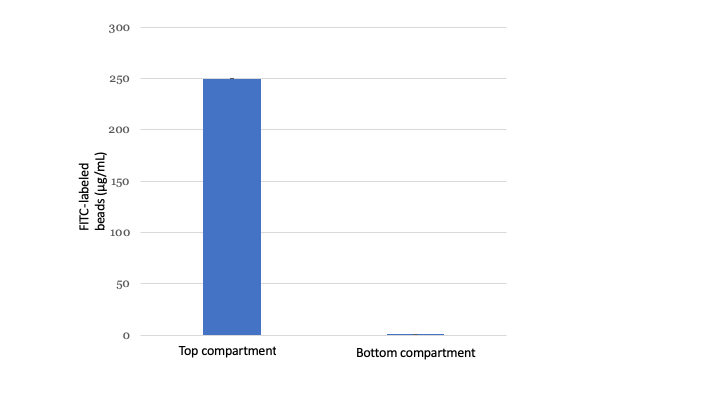

Supplement: S2 Fig — The monolayers excluded greater than 99.9% of the beads. (TIFF) [file pone.0226126.s002.tiff]
